# The Interactome between Metabolism and Gene Mutations in Myeloid Malignancies

**DOI:** 10.3390/ijms22063135

**Published:** 2021-03-19

**Authors:** Carmelo Gurnari, Simona Pagliuca, Valeria Visconte

**Affiliations:** 1 Department of Translational Hematology and Oncology Research, Taussig Cancer Institute, Cleveland Clinic, Cleveland, OH 44195, USA; carmelogurnari31@gmail.com (C.G.); paglius@ccf.org (S.P.); 2 Department of Biomedicine and Prevention, University of Rome Tor Vergata, 00133 Rome, Italy; 3 Immunology, Molecular Medicine and Applied Biotechnology, University of Rome Tor Vergata, 00133 Rome, Italy

**Keywords:** myeloid malignancies, *TET2* mutations, *IDH1*/*2* mutations, venetoclax, nicotinamide

## Abstract

The study of metabolic deregulation in myeloid malignancies has led to the investigation of metabolic-targeted therapies considering that cells undergoing leukemic transformation have excessive energy demands for growth and proliferation. However, the most difficult challenge in agents targeting metabolism is to determine a window of therapeutic opportunities between normal and neoplastic cells, considering that all or most of the metabolic pathways important for cancer ontogeny may also regulate physiological cell functions. Targeted therapies have used the properties of leukemic cells to produce altered metabolic products when mutated. This is the case of *IDH1*/*2* mutations generating the abnormal conversion of α-ketoglutarate (KG) to 2-hydroxyglutarate, an oncometabolite inhibiting KG-dependent enzymes, such as the *TET* family of genes (pivotal in characterizing leukemia cells either by mutations, e.g., *TET2*, or by altered expression, e.g., *TET1/2/3*). Additional observations derive from the high sensitivity of leukemic cells to oxidative phosphorylation and its amelioration using BCL-2 inhibitors (Venetoclax) or by disrupting the mitochondrial respiration. More recently, nicotinamide metabolism has been described to mediate resistance to Venetoclax in patients with acute myeloid leukemia. Herein, we will provide an overview of the latest research on the link between metabolic pathways interactome and leukemogenesis with a comprehensive analysis of the metabolic consequences of driver genetic lesions and exemplificative druggable pathways.

## 1. Introduction

During normal cellular development, metabolism is one of the principal modalities to produce energy. In fact, from cell birth to differentiation, cell metabolism, together with a switch in transcriptional factors and cytokine release in some instances, dictate cell fate. As an example, hematopoietic stem cells (HSCs) adapt the balance between survival and quiescence by keeping a low oxygen status, stabilizing the hypoxia-inducible factor (HIF-1α), and stimulate glycolysis activating enzymes regulating glucose uptake (glucose transporter 1, Glut1) and pyruvate synthesis (Lactate Dehydrogenase A, LDHA). The main demand of HSCs is maintaining themselves in a quiescent status which is easily reached by using low energetic power through glycolysis [1]. Studies have shown how changes in metabolism, specifically in the glycolytic process, are notably present in acute myeloid leukemia (AML) [2,3,4]. Indeed history goes back to the first measurements of tumor metabolism when Nobel Prize in Physiology and Medicine Otto Warburg discovered that malignant cells favor the production of lactic acid (anaerobic glycolysis) in the TCA cycle as a source of ATP production. Leukemia-initiating cells (LICs) have an increased glycolytic flux mediated by AMP-activated kinase (AMPK) activation and decreased levels of autophagic activity. Often, increased glycolytic activity has been associated with resistance to pharmacologic agents [5]. Moreover, the high flux of glucose has been linked to increased glucose-6-phosphate dehydrogenase (G6PD) and consequently to unfavorable prognosis. Indeed FLT3 inhibitors have been shown to inactivate G6PD in *FLT3* mutant AML [6]. Because of the high demand for glucose, frequently AML cells switch to the use of fructose as an energy source, suggesting that the inhibition of fructose uptake might be a way to starve AML cells and reduce their malignant potential.

Leukemia stem cells (LSC) rely mainly on oxidative phosphorylation (OXPHOS) through BCL-2, suggesting the basis to target BCL-2-dependent pathways [7]. Another modality of controlling LSCs to use OXPHOS is through glutamine metabolism. In fact, inhibition of the conversion of glutamine in glutamate by blocking glutaminases is able to arrest leukemic activity, and targeting glutamine conversion has been found efficient in synergy with BCL-2 inhibition [8]. Overproduction of endogenous reactive oxygen species is also another option to help AML cells to promote blast proliferation. Studies aiming to describe the global metabolic profiles and redox status of AML cells have shown differences in metabolic spectra between diverse cytogenetic and molecular subtypes [9,10]. Moreover, the presence of specific metabolites has been associated with prognosis and identified as markers of aggressiveness (phosphocholine and phosphoethanolamine) and chemoresistance (overexpression of glutathione). Altogether the deregulation of metabolic pathways in AML might contribute to therapy-resistance and relapse.

Targeting the metabolic pathway is feasible but mostly depends on a fine and specific targeting of only altered processes, given the importance of metabolic functions in normal cells. In this line, the concomitant use of metabolic pathways inhibitors and differentiation agents (BCL-2 inhibitors and hypomethylating agents, HMA) represents a promising avenue in drug discovery. Recently the combination of the data obtained from the Reactome and KEGG pathway and The Cancer Genome Atlas has defined the interaction between metabolic pathways and molecular and transcriptomic signatures in all cancers, including myeloid malignancies.

**Figure 1:**
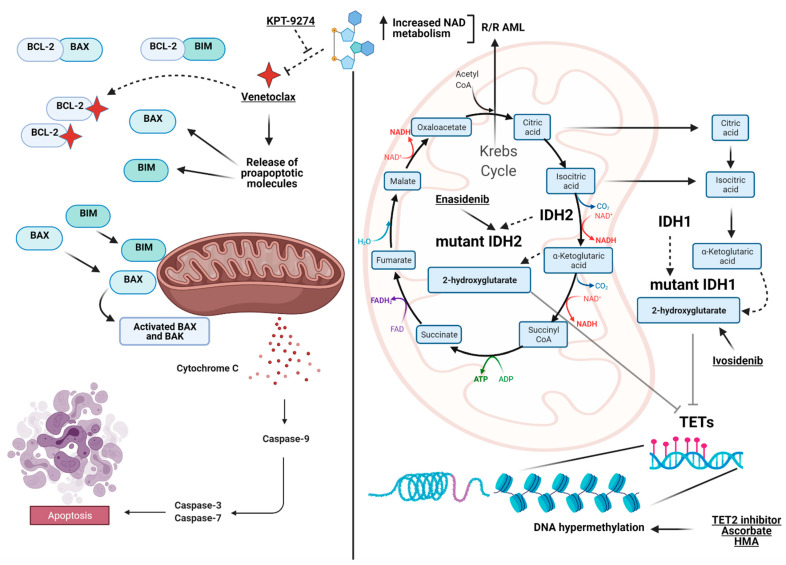
View at a glance of the landscape of the metabolic interactions between genes and pathways discussed in the manuscript. On the left, the intrinsic apoptotic pathway highlighted by BCL-2 as the pivotal player (targeted by Venetoclax) and the interaction with BAX and BIM to initiate the caspases cascade to trigger apoptosis. On the right, the interaction between the Krebs cycle (TCA), the convergence of IDH1/2 generating α-ketoglutarate, 2-hydroxyglutarate and the TET family of genes, which finally impacts DNA methylation. Included are also the available drugs used to target the depicted pathways. On the top, in relapsed/refractory (R/R) acute myeloid leukemia (AML), the interconnection between the increase in NAD metabolism generated by alteration of oxidative phosphorylation and Venetoclax resistance is shown together with proposed actionable target agents (e.g., KPT-9274) inhibiting Nicotinamide Phosphoribosyltransferase (NAMPT). Images were generated using BioRender.

**Figure 2:**
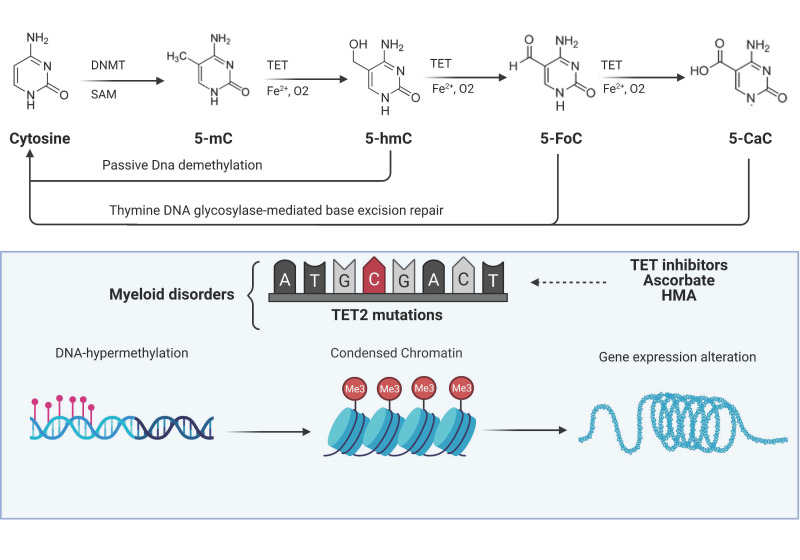
TET functions and their alterations in myeloid malignancies. DNA methyltransferases (DNMTs) initiate cytosine methylation with conversion to 5-methylcytosine (5-mc). TET proteins progressively oxidize 5-mC to 5-hydroxymethylcytosine (5-hmC), 5-formylcytosine (5-FoC), and 5-carboxylcytosine (5-CaC) creating a pool of TET-oxidized products (TDOP). 5-hmC can be reverted to cytosine via passive dilution while 5-FoC and 5-CaC via thymine DNA glycosylase-mediated base excision repair. Somatic *TET2* mutations create an imbalance in cellular DNA methylation through the disruption of the aforementioned mechanism with alteration of chromatin and thereby consequences on expression of genes regulating cell division and self-renewal. Images were generated using BioRender.

Here, we provide examples of the link between altered metabolism and leukemogenic potential, focusing on some of the major mutations in myeloid genes (e.g., *TET2* and *IDH1*/*2*) generating important biological consequences in a variety of cellular processes. Moreover, we will also review available therapeutic options and examples of dysregulation in genes involved in metabolism (Figure 1 and Figure 2).

## 2. The Metabolomics of TET Family of Genes

The Ten-Eleven Translocation (*TET*) gene family (*TET1*, *TET2*, and *TET3*) encodes for dioxygenase enzymes catalyzing the conversion of 5-methylcytosine (5-mC) to 5-hydroxymethylcytosine (5-hmC) [11]. The first discovered gene was *TET1*, identified as a partner of mixed-lineage leukemia (*MLL*/*KMT2A*) gene in a case of infant AML carrying a t(10:11) (q22;q23) translocation [12,13]. The finding of the dioxygenase function of TET1 led to the identification of the other two members of the TET family, TET2 and TET3 [14,15].

The carboxyl (C)-terminus of all three TET family members harbors the oxygenase catalytic domain and the binding sites for 2-oxoglutarate (α-KG) and Fe^2+^, which are both critical cofactors for TET oxidative activity [16]. TET proteins progressively oxidize 5-mC to 5-hmC, 5-formylcytosine (5-fC), and 5-carboxylcytosine (5-caC), creating a pool of TET-dependent 5-mC-DNA oxidation products (TDOP) which ultimately can be reverted to cytosine via thymine DNA glycosylase-mediated base excision repair [16,17].(Figure 2)

Therefore, TET proteins are critical regulators of DNA demethylation via the generation of 5-hmC, ultimately leading to enhanced gene expression and transcription profiles responsible for cell proliferation and survival [18,19,20]. As the role of the TET family of genes is highly crucial during cell development, any impairment either by genetic lesions or altered expression might impact the normal fate of the cells. Indeed, high levels of expression of TET family proteins and, as a consequence, of 5-hmC have also been found to be crucial for embryonic stem cells, determining cell fate and development with redundant functions [21,22]. As demonstrated by knockout (KO) experiments in mice, triple *Tet1/2/3* inactivation led to embryonic lethality while other *Tet1* and/or *Tet2* KO configurations were not completely disruptive [21,23,24]. TET2 haploinsufficiency altered HSC reprogramming into induced pluripotent stem cells, as demonstrated by an experiment of TET2 silencing via short hairpin RNA with different consequences according to the type of *TET2* mutation present [25]. Thus, while the pivotal role of *TET2* mutations is underlined by their high frequency in myeloid disorders, including AML, the lack of molecular lesions in the homologous *TET1/TET3* genes is probably explicable with a distinct tissue-specific expression and differences in the specific metabolic consequences derived from their imbalance [26].

### 2.1. TET2: A Pivotal Gene in Myeloid Malignancies

Somatic *TET2* mutations are commonly found in myeloid malignancies (MN) at frequencies varying according to disease subtypes. In myelodysplastic syndromes (MDS) and AML, *TET2* mutations are present in 20%–30% of cases, ranging up to 50% in patients affected by chronic myelomonocytic leukemia (CMML) [27,28,29]. Mutations are mainly loss-of-function (either frameshift or nonsense) affecting the coding region or missense mutations mapping in critical sites required for the enzymatic activity [30]. The prognostic role of *TET2* lesions has been controversial because of their high frequency, their heterogeneity, and the variability of the concurrent genetic lesions, all characteristics contributing to shaping the fate of individual patients and ultimately precluding a clear genotype/phenotype association [30]. Moreover, the discovery of mutations in myeloid genes in normal individual (referred to as CHIP, clonal hematopoiesis of indeterminate potential) at frequencies linearly correlated with age (age-related clonal hematopoiesis, ARCH) shed light on the process of myeloid evolution and provided clues on clonality and subclonal hierarchies in myeloid malignancies [31]. The occurrence of *TET2* mutations in CHIP/ARCH is another confirmation of the importance of this gene in cell development. Together with additional sex combs-like 1 (*ASXL1*) and DNA methyltransferase 3 alpha (*DNMT3A*) genes, *TET2* represents one of the most frequently mutated genes in CHIP/ARCH so that the three genes are referred to with the acronym DAT (*DNMT3A/ASXL1/TET2*) [31]. This finding, as well as the ubiquitous presence of *TET2* mutations in hematological malignancies, indicate that *TET2* lesions are mainly ancestral events occurring early in the course of the disease and contribute to the creation of a so-called “mutator phenotype”, by giving to the clone a proclivity for the acquisition of additional molecular lesions [32]. Indeed, our group demonstrated that in a cohort of 4930 patients with MNs, 1205 (24%) carried *TET2* mutations, which were ancestral and probably deriving from *TET2*-mutant CHIP in >40% of cases [30]. Subclonal acquisition of new leukemogenic events was identified as a facilitating condition for later myeloid progression and disease phenotypic determination (dysplastic vs. proliferative), as underlined by the higher number of secondary mutational events in *TET2*-mutant vs. *TET2* wild-type MN and in *Tet2* murine models [30]. Of note, progressive TET2 inactivation was associated with disease progression and poor survival outcomes as demonstrated by the aforementioned tendency of *TET2* mutant cases to accumulate additional hits in the same gene either resulting in biallelic mutations, deletions in hemizygous configurations, or uniparental disomies (UPDs) with homozygous mutations, with the latter two groups registering a negative impact on survival [30]. As a matter of fact, in another study focusing only on biallelic *TET2*-mutant MN, we confirmed that biallelic inactivation is frequently observed in MN and that this configuration is a typical feature of older patients with monocytosis (also outside the context of an overt CMML diagnosis), CMML, normal karyotype, and lower-risk disease. Therefore, biallelic *TET2* inactivation led to a disease phenotype skewed towards higher odds of monocytic vs. dysplastic features consistent with its prevalence in CMML [33]. Development of a CMML-like disease in mice has been reported in the literature, confirming the role of *TET2* in driving differentiation pressure towards a myelo-monocytic lineage consistent with the high frequency of *TET2* mutations in patients with CMML [34,35,36]. The association of *TET2* biallelic cases with lower-risk disease, rather than with a more aggressive phenotype as in the case of other *TET2* double-hits configurations (deletions in hemizygous configurations or UPDs with homozygous mutations), may be explainable with compensatory *TET1/TET3* stabilizing functions or acetylation mechanisms leading to increased TET2 protein stability [33]. The p300-mediated acetylation of key lysine residues (K110 and K111) at the amino (N)-terminus of TET2 enhances its functions and protects against DNA methylation during oxidative stress interacting with DNA methyltransferases (DNMTs) and regulating 5-mc/5-hmC balance [37]. The importance of metabolic events following *TET2* loss-of-function mutations in the pathogenesis of MN is also outlined by data showing its general down-regulation in patients with MN and the down-regulation of its family members [38]. Of note, low expression levels of the TET2 gene may be found independently of the presence of its mutations [39,40,41]. Indeed TET2 expression and 5-hmC levels were found also decreased in pediatric MDS cases, a population known to be rarely mutated in *TET2* gene [41]. TET2 expression levels have also been identified as a predictive and a prognostic biomarker in cytogenetically normal (CN)-AML [42]. Indeed, low TET2 expression had a negative impact on overall survival (OS) in both non-M3 and CN-AML (*p* = 0.016 and 0.044, respectively), although multivariable analysis confirmed these results only for the CN group [42]. Conversely, higher expression of TET1 at diagnosis was associated with poor clinical outcomes in a cohort of 360 CN-AML patients [43]. Finally, a recent study demonstrated that high TET3 expression was an independent factor for better OS and disease-free survival (DFS) in AML [44]. Of note, patients with lower TET3 expression undergoing hematopoietic stem cell transplant (HSCT) showed better OS and DFS than those who did not proceed to HSCT [44].

### 2.2. TET2 as an Actionable Target

Altogether, these data provide evidence that the TET family of genes, and in particular TET2, are actionable therapeutic targets in MN. In this line, recent findings suggested that ascorbic acid (AA) was able to restore some TET2 metabolic activities in vitro [45,46,47]. In addition, AA depletion in mice cooperated with *Flt3-ITD* mutations to accelerate leukemogenesis, whereas the reintroduction of dietary AA reversed this phenomenon by promoting Tet functions [46]. Moreover, AA was able to restore 5-hmC formation, drive DNA hypomethylation and expression of a TET2 gene signature, and ultimately suppressed leukemia progression in patient-derived xenografts (PDXs) [47]. Likewise, our group confirmed that long-term AA treatment prevented MN evolution in Tet2-deficient murine models [17]. However, TET2 loss due to catalytic domain lysine acetylation or missense mutations prevented this beneficial effect, which was restored by the additional use of class I and II histone deacetylase inhibitors [17]. Lower than normal AA levels have been found in patients with MN, and AA has been used at supraphysiological doses in a case of *TET2* mutant AML, confirming its potential therapeutic role in *TET2*-mutant MN [48]. Based on these considerations, many clinical trials (NCT03682029, NCT03999723) are trying to incorporate AA in the therapeutic schemes of MN, and future data will clarify the best settings for patients suitable for this treatment option [49].

Taking into consideration the biological consequences of *TET2* mutations, HMA constitutes a class of drugs currently available for patients with *TET2*-mutant MN. Studying a cohort of 213 MDS cases, Bejar et al. showed that *TET2* mutant patients had an increased likelihood of response to HMA treatment [50]. Similarly, other studies, including ours, described a better response to HMAs in patients harboring *TET2* mutations [51,52]. Indeed, as shown by competitive bone marrow transplantation experiments, HMA administration was able to significantly decrease *Tet2*-null cell proliferation advantage over wild-type cells (*p* = 0.002) [50].

Besides HMA, our group has recently developed a new therapeutic approach which entails the use of a TET-selective small-molecule inhibitor able to selectively suppress *TET2*-mutant cells in mouse models and *TET2*-mutated human leukemia xenografts while sparing normal cells [53].

Finally, in vitro and in vivo studies conducted in other models (hyperglycemic conditions) have depicted an impairment of the DNA 5-hydroxymethylome. TET2 has been identified as a substrate of the AMPK, which phosphorylates TET2 at serine 99. Increased glucose levels blocked AMPK-mediated phosphorylation at serine 99, causing the destabilization of TET2 followed by dysregulation of 5-hmC. This study also showed that administration of a biguanide (metformin) protected AMPK-mediated phosphorylation of serine 99 and increased TET2 stability and 5-hmC levels [54]. Similarly, studies conducted with AML have shown that AMPK is one of the major sensors of energy status and also for AML differentiation [55].

## 3. Isocitrate Dehydrogenase NADP(+) 1 and 2

*Isocitrate Dehydrogenase NADP(+) 1 and 2* genes encode enzymes of the tricarboxylic acid cycle (TCA), and mutations in these genes have been found to contribute to cancer development and progression because of the disruption of cell metabolism [56,57]. The two isoforms of *IDH1*/*2* metabolize isocitrate to α-KG in the mitochondrion (*IDH2*) as one of the steps of the Krebs cycle and in the cytoplasm (*IDH1*) [58]. Gain of function mutations in *IDH1* or *IDH2* result in a reduction in levels of α-KG and an increase in the formation of D-2-hydroxyglutarate (2-HG) [59,60]. α-KG represents one of the cofactors for several dioxygenases, such as the *TET* family of genes (see Section 2) and the Jumonji (Jmj) family of histone demethylases [61]. In general, α-KG is a crucial metabolic cofactor of histone and DNA demethylases, while 2-HG acts as a competitive inhibitor. The discovery of mutations of both *IDH* isoforms has led to the study of the interconnection between metabolism and epigenetics. *IDH* mutations are present in many cancers, including AML (around 20% of cases) [62]. Mutations are heterozygous and occur in substrate binding residues of *IDH1* (R132H) and *IDH2* (R140Q, R172K) [60]. Because of the nature of these mutations leading to the chemical formation of the oncometabolite 2-HG, several small-molecule inhibitors of the mutant form of both IDH have been developed and are showing good clinical responses [63,64]. Enasidenib and ivosidenib, IDH2 and IDH1 inhibitors respectively, were developed by Agios Pharmaceuticals and approved by the United States Food and Drug Administration (FDA) for the treatment of adult relapsed or refractory AML with *IDH2* and *IDH1* mutations [65,66]. Despite response rates at a frequency of about 50% achieved when used as monotherapy, a fraction of patients develop resistance to this class of agents. Resistance mechanisms have been attributed to the presence of co-occurring mutations in the *RAS*-family of genes (*FLT3*, *PTPN11*, and *KIT*), *RUNX1* and *CEBPA*, second site *IDH1* mutations, and isoform switching [67,68,69]. Therefore, a combination strategy with HMA has been envisioned as an option of investigation in AML patients ineligible for intensive chemotherapy (NCT02677922). Preclinical work has suggested that the addition of azacitidine (AZA) to the IDH1 inhibitor, ivosidenib, enhances mutant IDH1 inhibition-related differentiation and apoptosis. Indeed its combination resulted in a durable response, with the most complete responders showing a disappearance of *IDH1* mutations [70]. In fact, it was recently reported that the synergistic activity of the IDH1 inhibitor BAY1436032 with AZA in AML with *IDH1* mutations enhances anti-leukemia activity by co-targeting metabolism and methylation [71]. BAY1436032 showed profound antileukemic activity in two separate mutant AML derived xenografts, and it is now under clinical investigation for AML with *IDH1* mutations relapsed from or refractory to at least one previous line of therapy (NCT03127735). Inhibition of DNA methyltransferases and histones can also be achieved through loss-of-function mutations in the fumarate hydratase (FH) and succinate dehydrogenase (SDH) genes producing immoderate doses of fumarate and succinate, which act as competitive inhibitors of α-KG-dependent dioxygenases [72].

## 4. Venetoclax: An Agent Indirectly Inhibiting the TCA Cycle

The intrinsic apoptotic pathway consists of the B-cell lymphoma-2 (BCL-2) family, including proteins with conservative BCL-2–like homology domains 1–4 (BH1–BH4). These proteins are classified as suppressors (BCL-2/A1, BCL-XL/W, and MCL-1), activators (BIM and PUMA), effectors (BAX and BAK), and sensitizers (NOXA) [73]. The activation of the apoptotic pathway converges to some of these proteins, which ultimately create pores in the mitochondrial outer membrane with the release of cytochrome C and the activation of caspase 9, causing cell death (Figure 1, left panel). Inhibition of BCL-2 started several years ago with the discovery of the importance of this pathway in OXPHOS and metabolism of quiescent LSCs and the development of ABT-737, ABT-263 (navitoclax), and recently, GX15-070 (obatoclax) [74]. All these compounds were pan-inhibitors, with some causing thrombocytopenia as off-target effects involving BCL-XL and limited applicability to humans [75].

Venetoclax (ABT-199; VEN) is an oral BCL-2 selective BH3-mimetic agent which has been shown to inhibit mitochondrial metabolism by impairing TCA cycle activity, leading to the activation of reductive carboxylation [76,77]. As a result, VEN causes cellular metabolic reprogramming. Chemically this drug was developed to selectively target BCL-2, skipping the targeting of other homology BH family of proteins. BH3 profiling showed a positive correlation between VEN sensitivity and BCL-2 dependence, promoting the investigation of combinational strategy. Indeed, a number of agents have been combined with VEN, from daunorubicin to HMAs, neddylation inhibitors (MNL4924), FLT3 inhibitors (e.g., quizartinib), and IDH2 inhibitors (e.g., enasidenib) [78]. These studies led to the approval of VEN by the FDA in combination with HMAs (AZA or decitabine) or low-dose cytarabine as a front-line treatment in older patients with AML or unfit for induction chemotherapy [79]. VEN and HMAs, already possessing activity as single agents, showed higher rates of CR in combination in both treatment-naive and relapsed patients making this combo particularly promising. In particular, treatment with VEN and AZA led to superior clinical outcomes in older patients with AML [80]. Mechanistic studies showed that this treatment produces the inhibition of the electron transport chain complex II, disruption of the TCA cycle, and suppression of OXPHOS. Indeed OXPHOS is inhibited in vivo, and LSCs are eradicated in patients undergoing VEN/AZA treatment [81]. Additional confirmations derive from other data showing that the combination of the two drugs seems to decrease amino acid uptake, reducing OXPHOS and leading to LSC apoptosis [82]. However, VEN resistance has been linked to metabolic changes preventing cytochrome c release, and recent data on the co-targeting of mitochondrial complex I and BCL-2 showed promising results in AML cells reliant on OXPHOS [83]. In patients with R/R AML, the lower response to VEN/AZA has been correlated with increased levels of NAM and higher energy metabolism [81]. A schematic representation of the interplay between NAMPT, OXPHOS, and glycolysis pathways and their role in resistance to VEN is shown in Figure 1.

Di Nardo and colleagues’ data on VEN in combination with HMAs in the treatment- naïve elderly AML demonstrated that this combination was effective in most cytogenetic subgroups, including in patients harboring high-risk molecular features, *IDH1*/*2* lesions, and secondary AML [80,84]. However, ongoing studies are focusing on investigating the mechanisms behind the resistance of about a third of patients treated with VEN.

## 5. Nicotinamide: Pleyotropic Activity and Therapeutic Avenues

NAM adenine dinucleotide (NAD+) is an essential niacin-derived reduction-oxidation (redox) cofactor and cosubstrate for multiple enzymatic activities, playing a fundamental role in gene-regulatory, signaling, metabolic, and cellular homeostasis pathways, as well as in aging and diseases [85].

Our body is able to synthesize NAD+ from various dietary sources. A de novo pathway, starting from the metabolism of tryptophan degradation, uses quinolinic acid (QUIN). Others, described as salvage pathways, use dietary niacin (vitamin B3)—nicotinic acid (NA) and its pyridine-nucleoside or amide forms of nicotinamide riboside (NR) and nicotinamide (NAM), respectively. NAM is also produced from the reactions carried out by the enzymes that use NAD+ as a substrate, splitting the molecule at the N-glycosidic bond [86,87,88]. Three pathways have been identified for NAD+ utilization: the sirtuin (SIRT) mediated pathway and the ADP ribosylation—mono and poly-ribosylation—and cyclic ribosylation [89]. Within the mitochondria, NAD accepts electrons from a variety of sources and transfers them to complex I of the electron transport chain, ultimately resulting in the generation of ATP. NAD influences fuel selection, circadian rhythms, cell survival, and self-renewal under stress [90,91,92]. Although mechanisms of generating and maintaining the mitochondrial NAD pool are not completely understood, growing evidence shows that mitochondria not only can import NAD directly through an unrecognized membrane transporter but also have their specific nicotinamide mononucleotide adenylyltransferases (NMNATs) enzyme, able to convert nicotinamide mononucleotide (NMN) to NAD [93,94].

It is well established that HSCs tune cell cycle, apoptosis, and oxidative stress, which are at the basis of their balance among processes of quiescence, differentiation, and self-renewal. Deregulation of this balance can result in the development of blood disorders [95,96]. An interesting approach that is generating a growing body of evidence, both in vivo and in vitro, consists of using nicotinamide or NAD boosting agents as promoters of hematopoiesis [97,98,99,100]. The rationale for this approach is based on the stimulation of mitochondrial clearance in HSCs by increasing NAD+ content, a process at the basis of the self-renewal expansion supporting stress-induced hematopoiesis [101,102,103]. Mice supplemented with dietary NR boosted their hematopoietic progenitor compartments, showing a functional increase in short-term hematopoietic progenitors without affecting the self-renewing compartment of the long-term progenitors [97].

A recent phase I clinical trial has shown the efficacy of an expanded cord blood product (NiCord, NCT01816230) obtained after the supplementation of isolated CD133+ progenitors with nicotinamide and cytokines (including Flt3 ligand, stem cell factor, thrombopoietin, IL-6), infused after myeloablative conditioning [100]. This study showed significantly lower shorter neutrophil and platelet engraftment time compared to the non-expanded control group.

An ongoing phase I study (temporarily suspended during the COVID-19 pandemic), sponsored by the Case Comprehensive Cancer Center in Cleveland, aims to explore the role of NR in increasing donor-derived blood stem cell numbers in decreasing the engraftment time in other allogeneic stem cell transplant settings (NCT04332341).

Another interesting field of application in the setting of hematological neoplasms is the NAD+ capability of influencing the responsiveness to the cytotoxic effects elicited by DNA-damaging agents [104]. Genetic and pharmacological inhibition of NAM phosphoribosyltransferase (NAMPT), the rate-limiting enzyme in NAM metabolism, demonstrated selective eradication of relapsed LSCs. Indeed, it has been shown that VEN in combination with AZA failed to eradicate LSCs in R/R patients with AML (Figure 1) [105]. Metabolomic analysis revealed that elevated nicotinamide metabolism in relapsed LSCs activates amino acid metabolism and fatty acid oxidation, ultimately driving OXPHOS and blocking the apoptotic effect of both VEN and AZA [105]. A study conducted by high-throughput flow cytometry-based drug screen against cells of an AML case identified agents that selectively depleted the CD34+CD38- fraction (markers of LSCs), including four structurally-unrelated NAMPT inhibitors (FK866, STF-118804, GMX1778, and KPT-9274). Therefore, NAMPT inhibition represented an effective modality to selectively target human LSCs by decreasing intracellular NAD+ levels and inducing apoptosis (Figure 1) [106].

## 6. Other Gene Pathways Interconnected to Cell Metabolism

*Splicing factor 3b, subunit 1 (SF3B1).* About 20% of patients with MDS and 60%–82% of patients with MDS with ringed sideroblasts (RS) and MDS/myeloproliferative neoplasms with RS and thrombocytosis harbor mutations in the splicing factor 3b, subunit 1 (*SF3B1*) [107,108,109]. The *SF3B1* gene is a component of the RNA-splicing machinery and encodes a member of the U2 small nuclear ribonucleoprotein (U2snRNP), important in the branch point sequence recognition of pre-messenger RNA. A combination of transcriptomic and proteomic data has shown a number of metabolic genes altered in *SF3B1* mutant cells, such as a decrease in the mitochondrial complex III of the electron chain transport regulated by the ubiquinol-cytochrome c reductase complex assembly factor 1 (UBCC). Moreover, *SF3B1* mutant cells presented lower citric acid metabolites and abnormal splicing of mitochondrial enzymes (DLST). Interestingly, *SF3B1* mutant cells were characterized by missplicing and, therefore, deregulation of another metabolic gene, phoshoglycerase dehydrogenase (PHGDH), an enzyme controlling the nonessential amino acid serine. The exogenous supplementation of PHGDH was able to rescue the growth impairment of *SF3B1* mutant cells [110]. Drugs under investigation for patients with MN and splicing factors mutations are included in Table 1.

*FMS-like tyrosine kinase 3 (FLT3)*. *FLT3* mutations, mainly internal tandem duplications (*FLT3-ITD*), are found in 30% of AML patients and confer a poor prognosis and increased relapse rate. Studies conducted in murine cells have shown that cells overexpressing *FLT3-ITD* have increased glycolytic activity favoring ATP transfer from OXPHOS and promoting glycolysis [111]. Moreover, synthetic lethality screens conducted in human AML cells harboring *FLT3-ITD* mutations have found a number of metabolic genes (e.g., *ATM* and *G6PD*) able to sensitize *FLT3-ITD* cells to FLT3 inhibitors [6]. More recently, studies have shown that the combination of an FLT3 inhibitor (AC220) with OXPHOS inhibitors or glutaminase inhibitors was effective in models of *FLT3* mutant AML [112,113].

*MDS1 and EVI1 complex locus (MECOM alias EVI1).* The EVI1 gene encodes a transcriptional regulator and oncoprotein involved in hematopoiesis, apoptosis, and cell differentiation [114]. The protein represses the myeloid lineage-committed regulator *RUNX1* leading to the expression of creatine kinase mitochondrial 1 (CKMT1), which is involved in the conversion of arginine into creatinine. In xenograft models of AML expressing EVI1, inhibition of CKMT1 pharmacologically or via genetic knockout blocks ATP production and extends the life span of mutant mice [114].

All current therapeutic applications, their target genes, and mechanisms are shown in Table 1.

## 7. Conclusions

Myeloid malignancies present with a distinct metabolomic signature. Targeting metabolic pathways has become a potent therapeutic strategy for this group of disorders. The biological basis of this approach resides in the metabolic regulation of normal hematopoiesis and their alterations. Seminal studies (cited in this review) have described the minimal disturbances leading to altered metabolism. Current pharmacologic agents providing a window of response to patients with AML exemplify that targeting the metabolome is feasible, and in combination with currently approved agents, might increase the panoply of agents for patients failing first therapeutic approaches.

## Figures and Tables

**Table 1:** Therapeutic strategies, targets, and mechanisms in myeloid malignancies.

Metabolic Pathway	Genes Involved	Gene Mutation	Pathogenic Mechanisms	Drugs	Pharmacological Mechanism	Clinical Trial
**TET metabolism**	*TET1*, *TET2*, and *TET3*	*TET2*	Altered DNA-methylation	Bobcat339 and TETi76	TET2-mutant suppression	-
				Ascorbate	Restoration of 5-hmC formation, DNA-hypomethylation	NCT03682029 NCT03999723
				HMA *	DNA-hypomethylation	-
**TCA cycle**	*IDH1* and *IDH2*	*IDH1* and *IDH2*	Reduction in α-KG levels and increased 2-HG	Ivosidenib *	IDH1 inhibitor	NCT03839771
				Enasidenib *	IDH2 inhibitor	NCT03839771
				Venetoclax *	BCL-2 inhibitor	NCT04628026 NCT04628026
**Mitochondrial activity**	*SF3B1* and *PHGDH*	*SF3B1*	Abnormal splicing of mitochondrial enzymes	Phosphohydroxypyruvate	Serine synthesis pathway	-
				H3B-8800	Inhibition of early stages of spliceosome cascade	NCT02841540
				E7070	Intron retention and exon skipping	NCT01692197
**Creatine kinase pathway**	*EVI1*, *RUNX1*, and *CKMT1*	*MECOM*	Apoptosis and cell differentiation	Cyclocreatine	Inhibition of CKMT1	-
**OXPHOS**	*FLT3*	*FLT3*	OXPHOS/glycolysis imbalance	AC220 (Quizartinib)+ IACS-010759	Restoration of ATP levels	NCT02882321
**NAM metabolism**	Multiple genes encoding for enzymes of OXPHOS	NA	OXPHOS, amino acid metabolism alteration	OT-82, KPT-9274	NAMPT inhibitors	NCT03921879 NCT02702492

-, not available; α-KG, α-ketoglutarate; 2-HG, 2-hydroxyglutarate; BCL-2, B-cell lymphoma-2; OXPHOS, Oxidative phosphorylation; TET, Ten-Eleven Translocation 1,2,3; IDH, Isocitrate Dehydrogenase 1,2; SF3B1, Splicing factor 3b, subunit 1; PHGDG, phoshoglycerase dehydrogenase; MECOM, MDS1 and EVI1 complex locus; RUNX1, runt-related transcription factor 1; CKMT1, creatine kinase mitochondrial 1; FLT3, FMS-like tyrosine kinase 3. * These agents have been FDA-approved for clinical use.
